# Differential Effects of Concomitant Use of Vitamins C and E on Trophoblast Apoptosis and Autophagy between Normoxia and Hypoxia-Reoxygenation

**DOI:** 10.1371/journal.pone.0012202

**Published:** 2010-08-16

**Authors:** Tai-Ho Hung, Szu-Fu Chen, Meng-Jen Li, Yi-Lin Yeh, T'sang-T'ang Hsieh

**Affiliations:** 1 Department of Obstetrics and Gynecology, Chang Gung Memorial Hospital at Taipei, Taipei, Taiwan; 2 Department of Chinese Medicine, College of Medicine, Chang Gung University, Taoyuan, Taiwan; 3 Department of Physical Medicine and Rehabilitation, Cheng Hsin Rehabilitation Medical Center, Taipei, Taiwan; University of Sydney, Australia

## Abstract

**Background:**

Concomitant supplementation of vitamins C and E during pregnancy has been reportedly associated with low birth weight, the premature rupture of membranes and fetal loss or perinatal death in women at risk for preeclampsia; however, the cause is unknown. We surmise that hypoxia-reoxygenation (HR) within the intervillous space due to abnormal placentation is the mechanism and hypothesize that concomitant administration of aforementioned vitamin antioxidants detrimentally affects trophoblast cells during HR.

**Methodology/Principal Findings:**

Using villous explants, concomitant administration of 50 µM of vitamins C and E was observed to reduce apoptotic and autophagic changes in the trophoblast layer at normoxia (8% oxygen) but to cause more prominent apoptosis and autophagy during HR. Furthermore, increased levels of Bcl-2 and Bcl-xL in association with a decrease in the autophagy-related protein LC3-II were noted in cytotrophoblastic cells treated with vitamins C and E under standard culture conditions. In contrast, vitamin treatment decreased Bcl-2 and Bcl-xL as well as increased mitochondrial Bak and cytosolic LC3-II in cytotrophoblasts subjected to HR.

**Conclusions/Significance:**

Our results indicate that concomitant administration of vitamins C and E has differential effects on the changes of apoptosis, autophagy and the expression of Bcl-2 family of proteins in the trophoblasts between normoxia and HR. These changes may probably lead to the impairment of placental function and suboptimal growth of the fetus.

## Introduction

Oxidative stress has been considered to be an important intermediary step in the pathogenesis of preeclampsia [1]; however, several randomized clinical trials have failed to demonstrate any beneficial effect of concomitant supplementation of vitamin C (1000 mg) and vitamin E (400 IU) on the reduction of the rates of preeclampsia [2]–[7]. Instead, concomitant use of these antioxidants during pregnancy has been observed to increase the risk of low birth weight (less than 2500 g), preterm premature rupture of membranes (PROM), and fetal loss or perinatal death in women at risk for preeclampsia [3], [5], [7]. The reasons why concomitant supplementation of vitamins C and E differentially affects pregnancy outcomes in healthy pregnant women and women at risk for preeclampsia are unclear.

One of the most common characteristic features in placentas from pregnancies complicated by preeclampsia is insufficient trophoblast invasion of maternal endometrial spiral arteries [8]. As a result, perfusion of the placenta is impaired, and oxygen concentration within the intervillous space is more variable in comparison to a healthy pregnancy, resulting in an ischemia-reperfusion type injury [9]. Such a change in the prevailing oxygen concentration may lead to a difference in the susceptibility of cytotrophoblasts and syncytiotrophoblast for women with preeclampsia in comparison to those who have healthy pregnancies [10]. We have thus surmised that abnormal placentation is the cause of the differential effects of concomitant supplementation of vitamins C and E between women at risk for preeclampsia and healthy pregnant women.

Increased trophoblast apoptosis has been noted in placentas from pregnancies complicated by fetal growth restriction [11] and PROM [12], as compared to those from healthy pregnancies and in placentas from twin pregnancies with selective low birth weights [13]. Recent studies have also indicated that autophagy, an intracellular bulk degradation system responsible for lysosomal degradation of protein and other subcellular constituents, is increased in placentas from women with preeclampsia in comparison to those obtained from healthy pregnancies [14] and that it participates in the process of rupture of fetal membranes [15]. Autophagy and apoptosis are often co-activated in response to stress and both have been implicated in various physiologic or pathologic processes including cellular differentiation and cell death [16].

We previously established an *in vitro* hypoxia-reoxygenation (HR) model to study placental oxidative stress [17]. Using this model, we demonstrated that HR is a potent inducer of apoptosis in the human placenta and is a possible etiological factor in preeclampsia [18], [19]. More recently, we further showed that Bcl-2 family proteins, such as Bax and Bak, and mitochondrial oxidants are important in HR-induced trophoblast apoptosis [20]. Several studies from other organ systems have also indicated that Bcl-2 family proteins are involved in the regulation of autophagy [21].

Taken together, we hypothesized that concomitant administration of vitamins C and E differentially affects trophoblast apoptosis and autophagy between normoxia and HR conditions. These differential effects are mediated through the actions of Bcl-2 family proteins. The objectives of this study were as follows: (1) to investigate whether there is a differential impact of combined use of vitamins C and E on apoptosis and autophagy in the placenta between normoxia and HR, (2) to study the changes in the Bcl-2 family proteins, and (3) to explore the mechanism underlying the regulation of apoptosis and autophagy in trophoblast cells treated with vitamins C and E at normoxia and HR. We chose to study the effects of combined administration of vitamins C and E as they act synergistically *in vitro* [22] and are used in most clinical trials for prevention of preeclampsia [2]–[7].

## Materials and Methods

This study was approved by the Institutional Review Board of Chang Gung Memorial Hospital (No. 95-1392B and No. 95-1436B). All placental samples were obtained with written informed consent. The materials and chemicals used in this study were purchased from Sigma Chemical Co., St. Louis, MO, USA, except where other suppliers are stated individually.

### Placental tissue collection and culture

Human term placentas were obtained from normal pregnancies immediately after elective cesarean deliveries for repeat sections before the onset of labor. Villous samples (each about 50 mg wet weight) were taken midway between the chorionic and basal plates from lobules free of visible calcification or tears. After several brief rinses in ice-cold phosphate-buffered saline (PBS), villous samples were placed in a culture medium (Medium-199 with 25 mM HEPES, Earle's salts, and L-glutamine) equilibrated with 8% O_2_/5% CO_2_/balanced N_2_ in a sealed glass bottle and transferred to the laboratory on ice for individual experiments. Villous explants cultures were performed as previously detailed [20]. Briefly, villous samples were dissected into pieces weighing 5–10 mg in an ice-cold culture medium within a glove box equilibrated with 8% O_2_/5% CO_2_/balanced N_2_. Six to eight such pieces of villous tissue were suspended at the gas-liquid interface in individual Costar Netwell (24 mm in diameter, 500 µm mesh; Corning, NY, USA) supported in 3 ml of culture medium in individual controlled oxygen incubators (Forma Series II, Model 3130, Thermo Electron Corporation, Marietta, OH, USA; all of which were fully humidified) and maintained at 8% O_2_/5% CO_2_/balanced N_2_. The dissolved oxygen tension in the medium was continuously monitored with an oxygen meter (MI-730 Micro-Oxygen Electrode and OM-4 Oxygen Meter, Microelectrodes, Inc., Bedford, NH, USA).

After an overnight rest, the medium was changed, and villous explants were subjected to HR (8 hours at 2% oxygen, followed by 16 hours at 8% oxygen, two cycles) or kept at 8% oxygen throughout the experiment as the normoxic control. After 48 hours of incubation, villous explants were changed to fresh media equilibrated with 50 µM of ascorbic acid (dissolved in PBS) and 50 µM of α-tocopherol (dissolved in dimethyl sulfoxide) or with vehicles and kept at HR or normoxia. These doses have been used as the maximum plasma levels measured in a controlled trial of vitamins C and E supplementation for preventing preeclampsia [23]. Forty-eight hours later, the villous samples were briefly washed with PBS and snap-frozen in liquid nitrogen or fixed in 4% paraformaldehyde for further processing.

### Cytotrophoblasts isolation, purification, and culture

Cytotrophoblastic cells were isolated from term placentas as previously detailed [24]. Briefly, approximately 50 g of villous tissues were collected, finely minced, and dissociated in three 15-minute stages in Hank's balanced salt solution, 0.25% trypsin, and 300 U/ml DNase I. The resulting cell suspension was layered over a 5% to 70% discontinuous Percoll gradient (Roche Diagnostics GmbH, Mannheim, Germany) and centrifuged at 1200×g for 20 minutes. The cells migrating between the densities of 35% and 50% Percoll were collected, and then they were subjected to immunopurification by negative selection over columns consisting of magnetic microbeads coupled to mouse anti-human HLA class I antibody at a concentration of 40 µg/ml (clone W6/32; eBioscience, San Diego, CA, USA). Cells collected in this way are routinely >98% pure, as assessed by flow cytometric and immunohistochemical analysis for cytokeratin 7 (clone OV-TL12/30; DakoCytomation, Glostrup, Denmark). The purified cells were then plated at a minimum of 2×10^5^ cells/cm^2^ in 6-well plates and cultured under standard condition (5% CO_2_/balanced air) in Iscove's modified Dulbecco's medium with L-glutamine, 25 mM HEPES and 10% heat-inactivated fetal bovine serum.

After an overnight rest, the cells were rinsed twice with prewarmed culture medium to remove nonattached cells, and the medium was changed. The cells were then subjected to HR (8 hours at 2% oxygen, followed by 16 hours at standard culture condition, two cycles) or kept at standard culture conditions throughout as the normoxic control. After 48 hours of incubation, cells were changed to fresh media equilibrated with 50 µM of ascorbic acid and 50 µM of α-tocopherol or with vehicles and kept at HR or normoxia. Forty-eight hours later, cells were harvested for further processing.

### Assessment of villous apoptosis and autophagy

Apoptosis and autophagy of villous tissues were confirmed by transmission electron microscopy as previously described [18]. Apoptotic changes were further evaluated by a variety of techniques, including the terminal deoxynucleotidyl transferase (TdT)-mediated dUTP DNA-nick end labeling (TUNEL) assay, immunofluorescence for active caspase 3, immunohistochemistry for annexin IV, and the appearance of a cytokeratin 18 neoepitope (M30) produced downstream from caspase proteolytic action [18], [20]. We also applied immunohistochemistry to study the expression of autophagy-related proteins, LC3 and beclin-1.

Dual-color immunofluorescence against the active form of human caspase 3 and for the TUNEL assay was performed sequentially on the same sections from ten placentas, as previously described [18], [20]. Sections were mounted with Vectashield-DAPI (Vector Laboratories, Burlingame, CA, USA) and observed on a Leica TCS-SP2 confocal microscope (Leica Microsystems, Manheim, Germany). Negative controls were obtained by substitution of the primary antibody with non-immune rabbit IgG or by omission of the enzyme TdT. Staining for TUNEL and DAPI was examined sequentially at a magnification of 400×. Digital images from ten randomly selected fields that provided a minimum of 500 nuclei within the trophoblast layer (syncytiotrophoblast and cytotrophoblasts) for each section were saved. The percentage of nuclei that were stained by the TUNEL method divided by the total number of DAPI-stained nuclei was assessed.

### Immunohistochemistry

Immunohistochemistry for annexin IV, M30, LC3, and beclin-1 were carried out as previously described [20], [24]. After quenching endogenous peroxidase activity and blocking non-specific binding, sections were reacted with the primary antibodies listed in Table 1 at 4°C overnight. Further processing for colorimetric detection was performed according to the instructions for the Vectastain Elite ABC Kit (Vector Laboratories) using diaminobenzidine as the peroxidase substrate. Counter-staining with hematoxylin was carried out if needed. Negative controls included substitution of the primary antibody with non-immune goat or rabbit IgG or mouse isotypic IgG.

**Table 1:** Antibodies used in immunohistochemistry, immunofluorescence and Western blot.

Primary antibody	Commercial source	Catalog number	Species	Antibody type	Working concentration
Annexin IV (N-19)	Santa Cruz	sc-1930	goat	polyclonal	IHC 0.4 µg/ml
M30 (cytokeratin 18 neoepitope)	Roche	12-140-322-001	mouse	monoclonal, IgG_2b_	IHC 1∶100
Bcl-2	Dako	M0887	mouse	monoclonal, IgG_1κ_	IHC 1.125 µg/ml
Bcl-2 (N-19)	Santa Cruz	sc-492	rabbit	polyclonal	WB 2 µg/ml
Bcl-xL (H-5)	Santa Cruz	sc-8392	mouse	monoclonal, IgG_1_	IHC 2 µg/mlWB 2 µg/ml
Bax	Dako	A3533	rabbit	polyclonal	IHC 4.4 µg/mlWB 11 µg/ml
Bak (G-23)	Santa Cruz	sc-832	rabbit	polyclonal	WB 0.4 µg/ml
Bak	Upstate	06-532	rabbit	polyclonal	IHC 0.2 µg/ml
Active caspase 3	R&D	AF-835	rabbit	polyclonal	IF 250 ng/ml
LC3B	Cell Signaling	#2775	rabbit	polyclonal	WB 1∶200
LC3	MBL	PM036	rabbit	polyclonal	IHC 1∶1000
Beclin-1	Cell Signaling	#3738	rabbit	polyclonal	WB 1∶100
Beclin-1	MBL	PD017	rabbit	polyclonal	IHC 1∶200

IHC =  immunohistochemistry; IF =  immunofluorescence; WB =  Western blot.

Sections were viewed and photographed under a differential interference contrast microscope (Nikon Eclipse 80i, Nikon Corporation, Tokyo, Japan). With a 20× objective, five randomly selected fields for each section were examined by three investigators who were blinded to the identity of the tissue. The section was scored for intensity (absent, faint, moderate, or intense [0 through 3]) of M30 and annexin IV immunostaining in the trophoblast layer.

### Western blots

Western blots were performed as previously detailed [20], [24]. Fifty micrograms of either mitochondrial or cytosolic proteins was separated by 12% SDS-PAGE, transferred to nitrocellulose membranes, and probed with primary antibodies against human Bcl-2, Bcl-xL, Bax, Bak, LC3B, and beclin-1 (Table 1) at 4°C overnight. Cytosolic protein expression levels were normalized to β-actin (clone AC-15, 1∶5000 dilution; Sigma). Blots of mitochondrial proteins were confirmed for equal loading and transfer using MemCode reversible protein stains (Pierce Biotechnology, Rockford, IL, USA).

### Co-immunoprecipitation of Bcl-2, Bcl-xL and beclin-1

Co-immunoprecipitation was carried out as previously described [25]. After treatment in the HR condition, cytotrophoblast cells were lysed in a cold RIPA buffer (Pierce Biotechnology) containing a protease inhibitor cocktail (Roche). The lysate was centrifuged and protein concentration determined (Bio-Rad, Hercules, CA, USA). Crude lysates were precleared with protein A/G plus-agarose (Santa Cruz Biotechonology, Inc. Santa Cruz, CA, USA) and incubated with anti-beclin-1 (1∶50) at 4°C overnight. Immune complexes were precipitated with protein A/G plus-agarose beads followed by two washes in RIPA buffer. The pellet was resuspended in a 2x Laemmli sample buffer and proteins were resolved by SDS-PAGE and immunoblotted forBcl-2 and Bcl-xL as above.

### Real-time quantitative PCR

Total RNA was extracted from cytotrophoblastic cells using RNeasy Mini Kits (Qiagen, Valencia, CA, USA) and then subjected to reverse transcription using SuperScript II RNase H reverse transcriptase (Invitrogen, Carlsbad, CA, USA). Real-time quantitative PCR analysis was performed with an ABI PRISM 7900 sequence detector (Applied Biosystems, Foster City, CA, USA). Assay-on-Demand TaqMan primers and probes from Applied Biosystems were used. These included Bcl-2 (TaqMan Gene Expression Assay ID Hs00608023_m1), Bcl-xL (Hs00236329_m1), Bax (Hs00751844_s1), Bak (Hs00832876_g1), LC3A (Hs00738808_m1), LC3B (Hs00797944_s1), LC3C (Hs01374916_m1) and beclin-1 (Hs00186838_m1). 18S ribosomal RNA (Hs99999901_s1) was used as an endogenous control. Thermal cycling was initiated with a 2-minute incubation at 50°C, followed by a first denaturation step of 10 minutes at 95°C, and then 40 cycles of 95°C for 15 seconds and 60°C for 1 minute. All samples were analyzed on the same run, and each sample was run in triplicate. Relative quantities of Bcl-2, Bcl-xL, Bax, Bak, LC3A, LC3B, LC3C and beclin-1 mRNA, and 18S ribosomal RNA were calculated by the comparative threshold cycle (Ct) method as previously described [20].

### Statistical analysis

Data are presented as mean ± SEM or median and interquartile range. Differences between the vehicle control and vitamin treatment group were computed with the Student's *t*-test or Mann-Whitney *U*-test. Statistical significance was set at *P*<0.05.

## Results

### Decreased apoptotic changes in villous explants treated with vitamins C and E at normoxia (8% O_2_)

**Figure 1:**
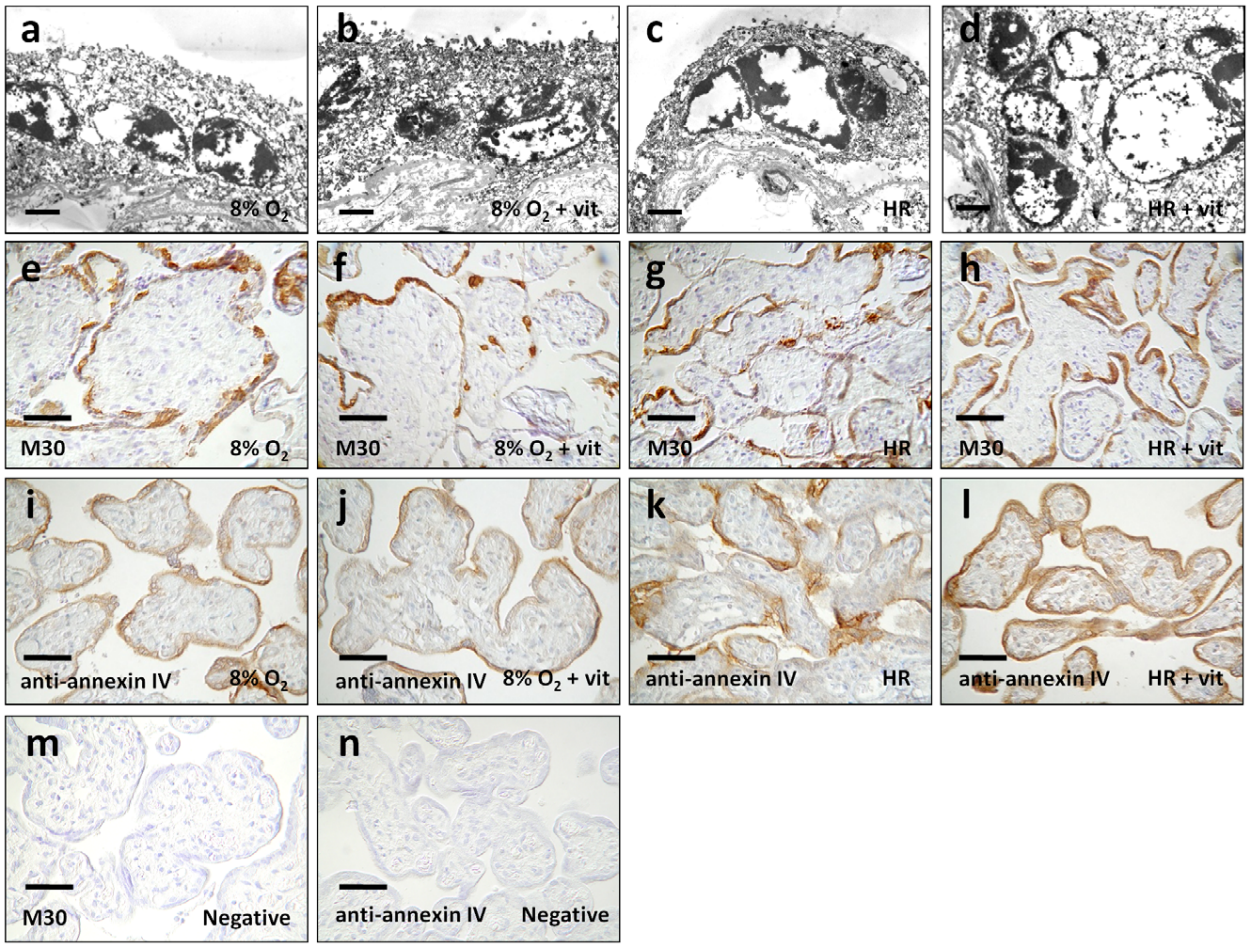
Apoptotic changes in villous explants. (a–d) Electron micrographs illustrating apoptotic changes, including shrinkage of the nuclei, chromatin condensation, and nuclear fragmentation in the trophoblast layer of the villous tissues in all experimental conditions. (e, f, i, j) Under 8% oxygen, less immunoreactivity of annexin IV was noted in villous tissues treated with vitamins C and E in comparison to the vehicle controls. There was, however, no significant difference in the intensity of M30 immunostaining between the two groups. (g, h, k, l) In contrast, more prominent and extensive immunostaining of M30 and annexin IV was noted in villous tissues treated with vitamins C and E at HR conditions. (m, n) Substitution of the primary antibody with non-immune mouse IgG_2b_ (for M30) and goat IgG (for annexin IV) served as the negative controls. Scale bar = 1 µm (a–d) and 50 µm (e–n).

**Figure 2:**
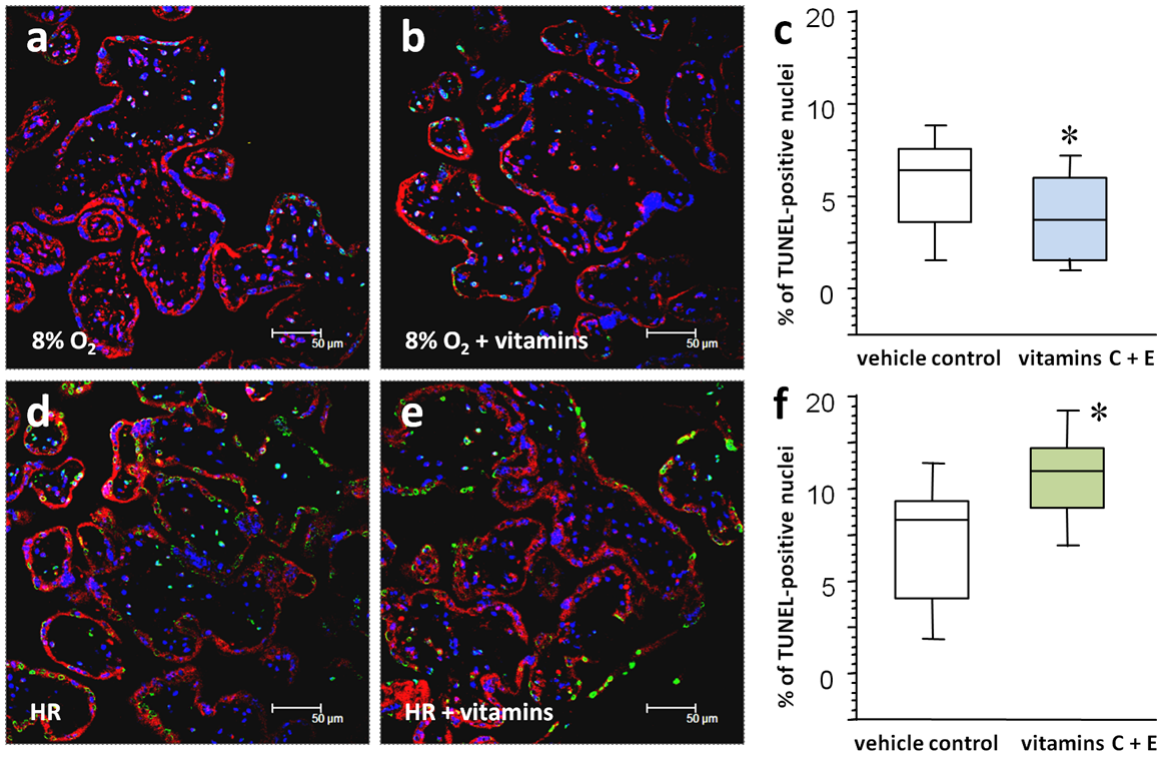
Immunofluorescent labeling for active caspase 3 and TUNEL assay. (a–c) Administration of vitamins C and E reduced the number of TUNEL-positive nuclei (green) and less immunofluorescence of active caspase 3 (red) in the trophoblast layer of villous tissues kept at 8% oxygen throughout the experiment. (d–f) On the other hand, vitamin treatment caused more TUNEL-positive nuclei and activation of caspase 3 in the trophoblast layer in comparison to the vehicle controls in villous tissues subjected to HR. Sections were stained with DAPI to show all nuclei. Central bars represent the median values, boxes represent the interquartile ranges, and whiskers represent the 90th and 10th percentiles for ten separate experiments. *, *P*<0.05, in comparison to vehicle controls.

Using electron microscopy, apoptotic changes were clearly demonstrated in the trophoblast layer of the villous tissues in all experimental conditions. These included shrinkage of the nuclei, chromatin condensation, and nuclear fragmentation (Figures 1a–1d). We also used immunohistochemistry to detect annexin IV and cleavage of cytokeratin 18 as markers of trophoblast apoptosis. Under 8% oxygen, there was less immunoreactivity of annexin IV in villous tissues treated with vitamins C and E when compared to the vehicle controls (Figures 1i, 1j and Table 2). Likewise, administration of vitamins C and E reduced the number of TUNEL-positive nuclei (3.5% in the vitamin group versus 6.5% in the control group, *P*<0.05), and there was less immunofluorescence of active caspase 3 in the trophoblast layer of villous tissues kept at 8% oxygen throughout (Figures 2a–2c). There was, however, no significant difference in the intensity of M30 immunostaining between the two groups (Figures 1e, 1f and Table 2).

**Table 2:** Intensity of M30 and annexin IV immunostaining in villous tissues at normoxia (8% O_2_) and HR.

	8% O_2_		*P*	HR		*P*
	Controls	Vit. C+E		Controls	Vit. C+E	
M30	1.1±0.2	0.6±0.1	0.147	1.7±0.1	2.3±0.2	0.018
Annexin IV	1.4±0.1	0.6±0.2	0.002	1.7±0.1	2.5±0.2	0.001

Data presented as mean ± SEM.

*P* values based on Student's *t*-test.

The intensity of staining was scored as 0 (absent), 1 (faint), 2 (moderate), or 3 (intense).

### Increased apoptotic changes in villous explants treated with vitamins C and E at HR

In contrast, more prominent and extensive immunostaining of M30 and annexin IV was noted in villous tissues treated with vitamins C and E in HR conditions (Figures 1g, 1h, 1k, 1l and Table 2). There were also more TUNEL-positive nuclei (11.5% in the vitamin group versus 8.5% in the control group, *P*<0.05) and activation of caspase 3 in the trophoblast layer in comparison to the vehicle controls (Figures 2d–2f). We further compared the percentage of TUNEL-positive nuclei between the four groups using Friedman test for matched non-parametric data and confirmed the difference between the control group and the vitamin group either at normoxia or HR. However, there was no significant difference in the percentage of TUNEL-positive nuclei between control groups of normoxia and HR, as considerable inter-individual variations were noted.

### Differential changes in autophagy in villous explants treated with vitamins C and E between normoxia (8% O_2_) and HR

**Figure 3:**
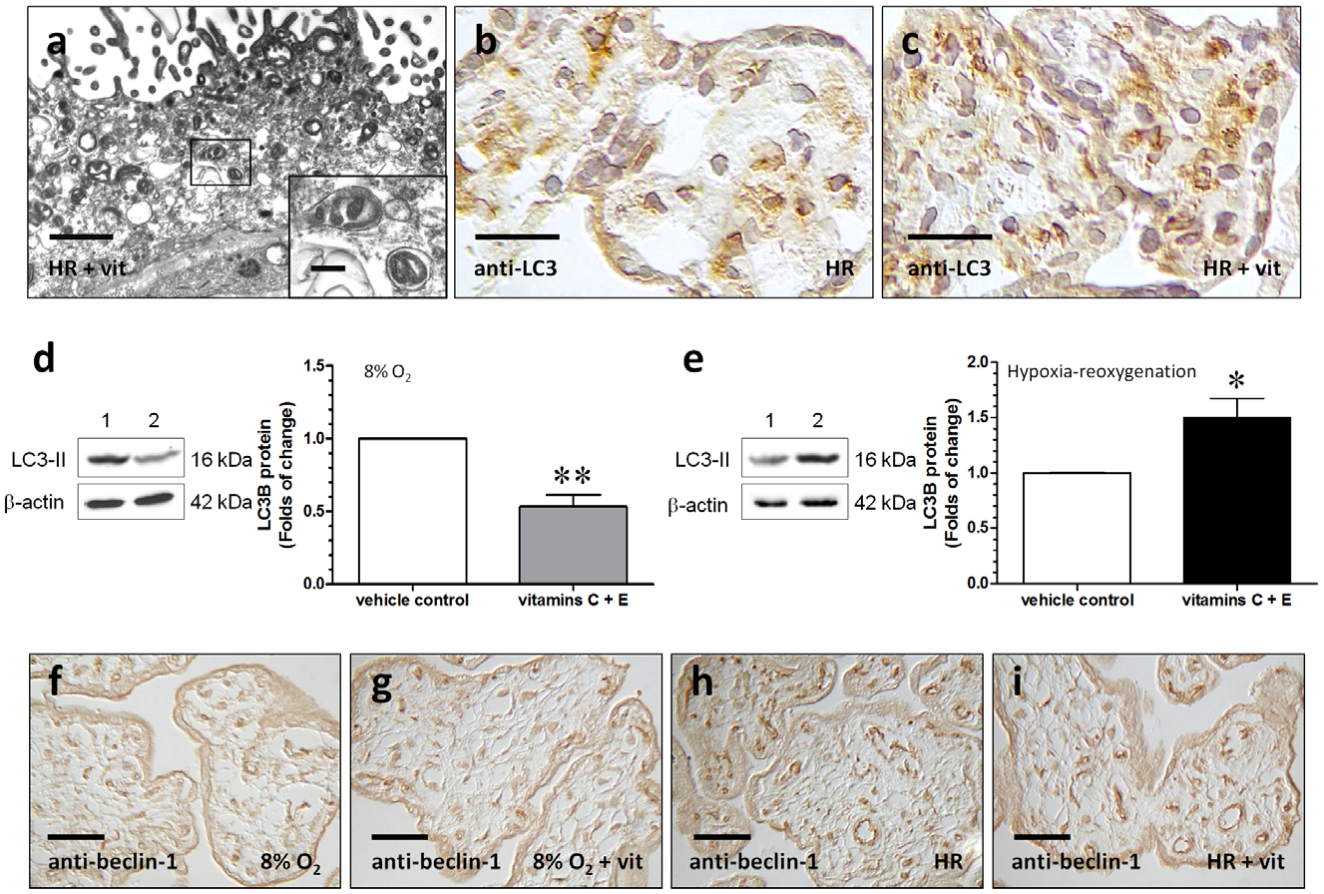
Autophagic changes in villous explants. (a) Electron micrographs illustrating autophagic vacuoles in the trophoblast layer of the villous tissues treated with vitamins C and E under HR condition. These autophagic vacuoles contain intracytoplasmic organelles, such as mitochondria. Scale bar = 1 µm and 200 nm (insets). (b, c) Vitamin treatment led to more prominent staining of LC3 in the trophoblast layer and some stromal cells in villous tissues subjected to HR. Scale bar = 20 µm (d, e) Using an antibody with a stronger reactivity to the type II form of LC3B to study the autophagic changes, a decrease in the level of LC3-II was noted in villous explants treated with vitamins C and E at 8% oxygen in comparison to the vehicle control. In contrast, the amount of LC3-II was increased in explants treated with vitamins C and E at HR. Lane 1, vehicle control and lane 2, vitamin treatment group. Data are presented as mean ± SEM for 10 separate experiments. *, *P*<0.05 and **, *P*<0.01, in comparison to vehicle controls. (f–i) There was no difference in the staining of beclin-1 between the vehicle control and the vitamin treatment group at either 8% oxygen or HR. Scale bar = 50 µm.

By using electron microscopy, autophagic vacuoles were observed in the trophoblast layer. These autophagic vacuoles contain intracytoplasmic organelles, such as mitochondria (Figures 3a). Under normoxic conditions, the administration of vitamins C and E reduced the intensity of immunostaining of LC3 in comparison to the vehicle control (data not shown). However, vitamin treatment led to more prominent staining of LC3 in the trophoblast layer and some stromal cells in villous tissues subjected to HR (Figures 3b, 3c). This was further confirmed by Western blots, using an antibody that detects endogenous levels of total LC3B protein and has a stronger reactivity with the type II form of LC3B (Figures 3d, 3e). There was no difference in the staining of beclin-1 between the vehicle control and the vitamin treatment group, either at 8% oxygen or HR (Figures 3f–3i).

### Increased Bcl-2 and Bcl-xL and decreased LC3-II in cytotrophoblastic cells treated with vitamins C and E at standard culture condition

**Figure 4:**
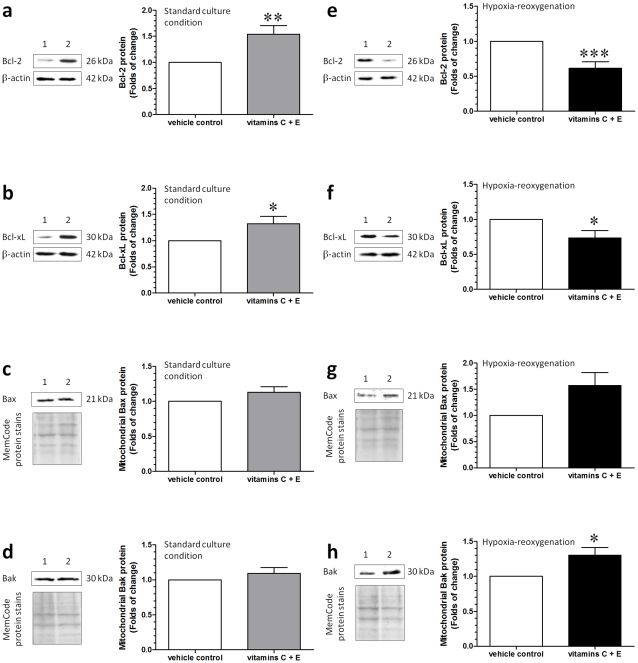
Changes in Bcl-2, Bcl-xL and mitochondrial Bax, and Bak in cytotrophoblastic cells. (a–d) In comparison to the vehicle control, increased levels of anti-apoptotic proteins, Bcl-2 and Bcl-xL, were noted in cytotrophoblastic cells treated with vitamins C and E at standard culture conditions. There was no difference in the levels of mitochondrial pro-apoptotic proteins, Bax and Bak, between the two groups. (e-h) In contrast, a decrease in the levels of Bcl-2 and Bcl-xL and an increase in the mitochondrial Bak protein levels were noted in cytotrophoblastic cells treated with vitamins C and E at HR in comparison to the vehicle control. Lane 1, vehicle control and lane 2, vitamin treatment group. Data are presented as mean ± SEM for 12 separate experiments. *, *P*<0.05; **, *P*<0.01 and ***, *P*<0.001, in comparison to vehicle controls.

Since the trophoblast layer is the functional layer of the human placenta and displayed the most prominent apoptotic and autophagic changes, we isolated the cytotrophoblastic cells from the term placentas to study the role of the Bcl-2 family proteins in the regulation of these changes. In comparison to the vehicle control group, increased levels of Bcl-2 and Bcl-xL were noted in cytotrophoblastic cells treated with vitamins C and E in standard culture conditions (Figures 4a, 4b). There were no differences in the levels of mitochondrial Bax and Bak between the two groups (Figures 4c, 4d).

**Figure 5:**
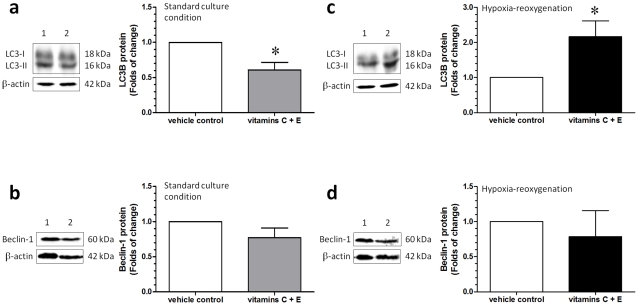
Changes of LC3-I, LC3-II and beclin-1 in cytotrophoblastic cells. (a, c) In comparison to the vehicle control, a decrease in the level of LC3-II was noted in cytotrophoblastic cells treated with vitamins C and E at standard culture conditions. In contrast, the relative amount of LC3-II was increased in cytotrophoblastic cells treated with vitamins C and E at HR. (b, d) However, there were no differences in the levels of LC3-I and beclin-1 between the vehicle control and vitamin treatment groups at either standard culture conditions or HR. Lane 1, vehicle control and lane 2, vitamin treatment group. Data are presented as mean ± SEM for 12 separate experiments. *, *P*<0.05, in comparison to vehicle controls.

After synthesis, LC3 is processed to its cytosolic form, LC3-I, and modified to the active form LC3-II. LC3-II then binds to the outer membrane of autophagosomes. Thus, the relative amount of LC3-II reflects autophagic activity. As shown in Figure 5a, there was a decrease in the level of LC3-II in cytotrophoblastic cells treated with vitamins C and E in the standard culture condition; however, there was no difference in the level of beclin-1 between the two groups (Figure 5b).

### Decreased Bcl-2 and Bcl-xL and increased mitochondrial Bak and cytosolic LC3-II in cytotrophoblastic cells treated with vitamins C and E at HR

In contrast, a decrease in the levels of Bcl-2 and Bcl-xL (Figures 4e, 4f) and an increase in the mitochondrial Bak (Figure 4h) protein levels were noted in cytotrophoblastic cells treated with vitamins C and E at HR. These changes were associated with an increase in the level of LC3-II (Figure 5c); however, there were no differences in the levels of beclin-1 (Figure 5d) and mitochondrial Bax (Figure 4g) between the vehicle control and the vitamin treatment group at HR.

### Vitamin treatment reduced the interactions of beclin-1 with Bcl-2 and Bcl-xL at HR

**Figure 6:**
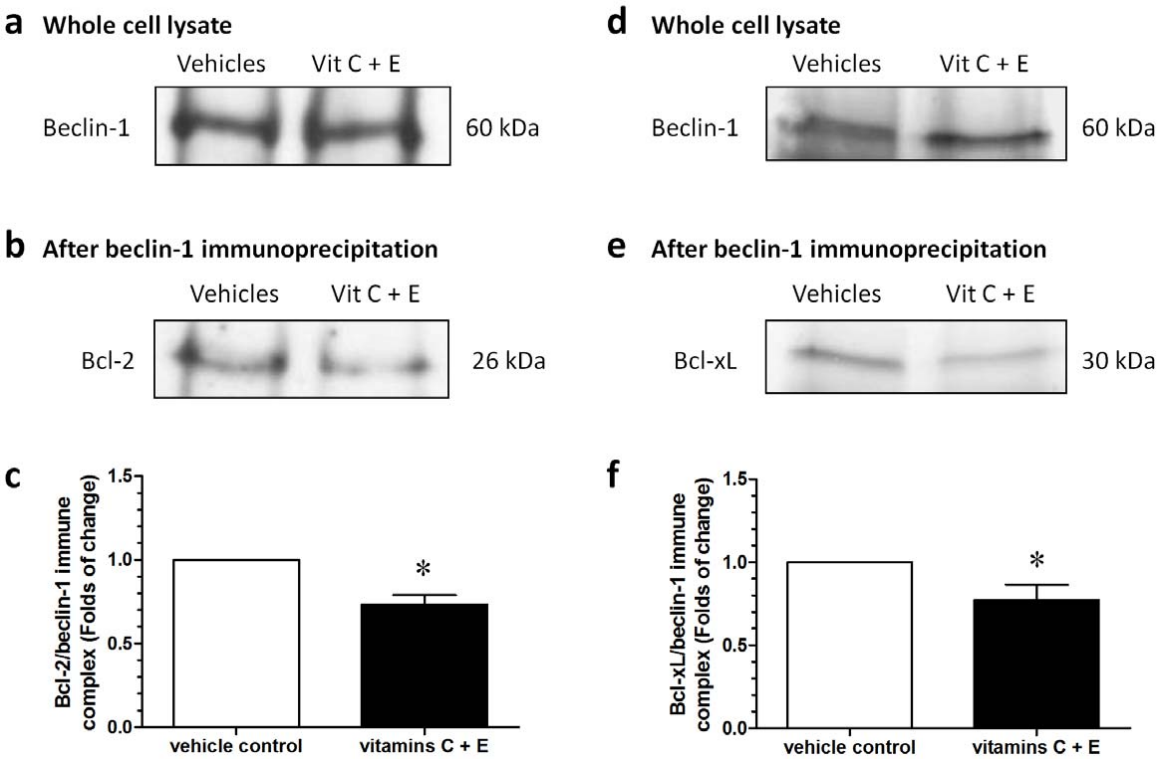
Co-immunoprecipitation experiments to examine the interactions of beclin-1 with Bcl-2 and Bcl-xL. Cytotrophoblastic cells treated with vitamins C and E or vehicles were subjected to HR conditions, and lysates were immunoprecipitated with an antibody to beclin-1 before immunoblotting for Bcl-2 or Bcl-xL. (a, d) There was no difference in the amount of beclin-1 in the whole cell lysates. (b, c, e, f) The vitamin treatment was associated with decreased amounts of immune complex of Bcl-2/beclin-1 and Bcl-xL/beclin-1. Data are presented as mean ± SEM for 5 separate experiments. *, *P*<0.05, in comparison to vehicle controls.

We conducted co-immunoprecipitation experiments to examine the interactions of beclin-1 with Bcl-2 and Bcl-xL. Cytotrophoblastic cells treated with vitamins C and E or vehicles were subjected to the HR condition, and lysates were immunoprecipitated with an antibody to beclin-1 before immunoblotting for Bcl-2 and Bcl-xL. There was no difference in the amount of beclin-1 in the whole cell lysates between the two groups (Figures 6a, 6d). However, the vitamin treatment was associated with decreased amounts of immune complex of Bcl-2/beclin-1 (Figures 6b, 6c) and Bcl-xL/beclin-1 (Figures 6e, 6f).

### Vitamin treatment led to differential changes in the transcription of Bcl-2, Bcl-xL, and Bak between standard culture conditions and HR

**Figure 7:**
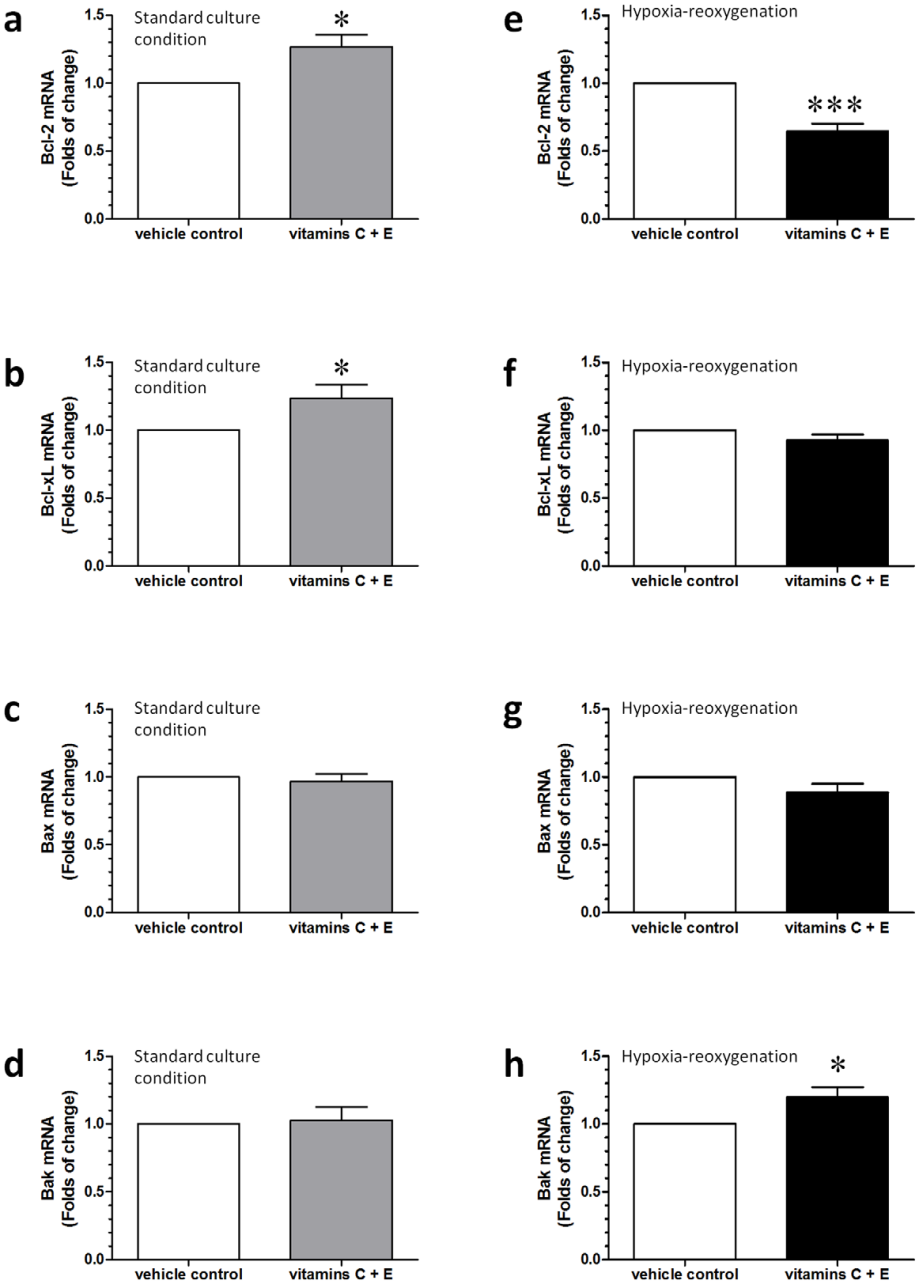
Differential changes in the transcription of Bcl-2, Bcl-xL, Bax and Bak. (a–d) By using real-time quantitative PCR, increased expression of Bcl-2 and Bcl-xL mRNA was noted in cytotrophoblastic cells treated with vitamins C and E at standard culture conditions. (e–h) In contrast, vitamin treatment decreased the expression of Bcl-2 mRNA and increased the expression of Bak mRNA in comparison to the vehicle control in cytotrophoblastic cells subjected to HR. Data are presented as mean ± SEM for 12 separate experiments. *, *P*<0.05; ***, *P*<0.001, in comparison to vehicle controls.

**Figure 8:**
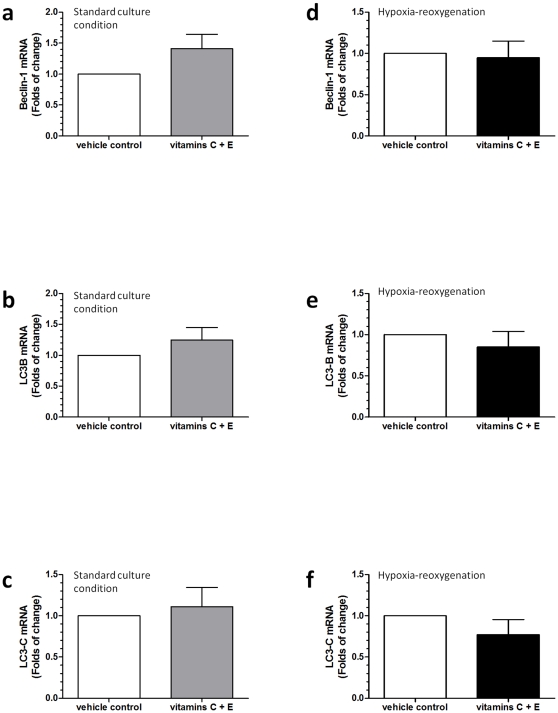
Differential changes in the transcription of LC3 and beclin-1. There were no differences in the levels of transcription of LC3B, LC3C, and beclin-1 between the vehicle control and the vitamin treatment groups either at standard culture conditions (a–c) or HR (d–f). Data are presented as mean ± SEM for 12 separate experiments.

We next investigated changes in the transcriptions of these proteins. By using real-time quantitative PCR, increased expressions of Bcl-2 and Bcl-xL mRNA were noted in cytotrophoblastic cells treated with vitamins C and E at standard culture conditions (Figures 7a–7d). In contrast, vitamin treatment decreased the expression of Bcl-2 mRNA and increased the expression of Bak mRNA in comparison to the vehicle control in cytotrophoblastic cells subjected to HR (Figures 7e–7h). There was no difference in the levels of transcription of LC3B, LC3C, and beclin-1 between the vehicle control and the vitamin treatment groups, either at standard culture conditions or HR (Figure 8). Noticeably, we were not able to detect the expression of LC3A mRNA in cytotrophoblastic cells.

## Discussion

The ineffectiveness of vitamins C and E in the prevention of preeclampsia and the potentially harmful effects disclosed by several clinical trials emphasize the need for a better understanding of the underlying mechanisms and metabolism of these antioxidant vitamins in pregnant women. By using villous explants, we found that the concomitant administration of vitamins C and E decreased the apoptotic and autophagic changes in the trophoblast layer at normoxia, but caused more prominent apoptosis and autophagy in the trophoblast layer at HR. We also demonstrated increased levels of Bcl-2 and Bcl-xL in cytotrophoblastic cells treated with vitamins C and E at standard culture conditions. These changes were associated with a decreased level of LC3-II, reflecting a reduction in autophagic activity. On the other hand, the concomitant administration of vitamins C and E decreased Bcl-2 and Bcl-xL levels and increased mitochondrial Bak levels, in addition to increasing of LC3-II in cytotrophoblasts subjected to HR. Together, these results indicate that concomitant administration of vitamins C and E has differential effects on the changes of apoptosis, autophagy and the expression of Bcl-2 family of proteins in the trophoblasts between normoxia and HR.

It is arguable that there may have been harmful effects on the variability and function of trophoblast cells during the nearly 5 days of experiments in this study. Indeed, degeneration of the syncytiotrophoblast is noted in third-trimester explants after 8 hours of culture; however, a newly formed trophoblast layer is found by 48 hours [26], [27]. By studying the transport activity, secretion of human chorionic gonadotrophin (hCG) and ultrastructural changes, Siman and colleagues showed that trophoblast cells in villous explants degenerate during the first 2 days but improve their integrity and responsiveness with time in culture over the 7 days studied, suggesting that the placental tissue is able to repair the severe trauma caused by preparation of the culture system [26]. Later, Crocker et al. exposed term villous tissues to different oxygen concentrations (3% and 17% O_2_) or tumor necrosis factor (TNF)-alpha and extended the culture period to 11 days for functional and morphological assessment [27]. They confirmed that explant cultures with a longer incubation period are valuable for studying the death and repair of the trophoblast and for investigating the chronic regulation of placental function under pathological conditions. Based on these observations, we thus carried out our experiments for 5 days to investigate the chronic effects of vitamins C and E on trophoblast cells in response to oxidative stress.

In addition to allowing regeneration of syncytiotrophoblast and investigating chronic regulation of apoptosis and autophagy, another reason for us to incubate villous explants and cytotrophoblastic cells under normoxia or HR for 48 hours before administration of vitamins C and E is to precondition the trophoblast cells with our experimental settings. It has been demonstrated that isolated cytotrophoblasts from preeclampsia or fetal growth restriction and their derived syncytiotrophoblast are more sensitive to apoptosis compared with those from normal pregnancies [10]. These results suggest that there are intrinsic variations in the cellular reactivity of trophoblasts, probably caused by abnormal placentation, between normal pregnancies and pregnancy complications such as preeclampsia. Therefore, we feel that preconditioning the villous explants or cytotrophoblastic cells to HR for a period before administration of vitamins C and E is important in investigating the effects of these antioxidant vitamins. Taken together, we believe our experimental settings more closely reflect real situations, as women start to take vitamins C and E several weeks after placentation and continue vitamin supplementation for several months of gestation.

The reasons why concomitant supplementation of vitamins C and E during pregnancy has different effects on pregnancy outcomes between healthy pregnant women and women at risk for preeclampsia remain unclear. Based on our observations, we propose a possible mechanism to explain the detrimental effect of the concomitant use of vitamins C and E on trophoblast cell death. In healthy pregnant women, perfusion of the intervillous space is relatively constant. Under this condition, vitamins C and E increase the amounts of Bcl-2 and Bcl-xL, which may inhibit the actions of Bax and Bak, thus reducing apoptosis. Bcl-2 and Bcl-xL can also bind to beclin-1, as revealed by our co-immunoprecipitation experiments, preventing its activation on the autophagy-inducing lipid kinase, Vps34, and subsequent autophagy [21]. However, under HR conditions, which we believe reflect the situation within the placentas of women at risk for preeclampsia, vitamin treatment decreases the amount of Bcl-2 and Bcl-xL, leading to more Bax and Bak present in the mitochondrial membrane to form transition pores with the subsequent release of cytochrome *c* and the activation of caspase 3. Although we noted no changes in the levels of beclin-1 in this study, reduced levels of Bcl-2 and Bcl-xL indicate more beclin-1 is available to activate the enzyme Vps34 and to induce the formation of autophagosomes. Increased apoptosis in the trophoblasts may contribute to impaired placental function and suboptimal fetal growth.

The significance of the increased autophagy in trophoblastic cells displaying apoptotic changes is not yet known, though the activation of autophagy has been shown to be a cellular survival strategy in other organ systems [16]. In addition to maintaining cellular energy homeostasis during starvation, autophagy is also involved in eliminating damaged mitochondria and other organelles and in degrading misfolded proteins and is thus a clean-up or self-clearance mechanism in cells committed to die by apoptosis or necrosis [16]. Our previous work showed that the Bcl-2 family of proteins in the outer mitochondrial membrane modulates HR-induced apoptosis [20]. Here, we further demonstrated that the Bcl-2 family of proteins is involved in the regulation of autophagy. These results suggest that mitochondria represent a nexus at which the autophagy and apoptosis pathways may interact. Further studies are being carried out to investigate how autophagy affects the trophoblast cell fate under HR-induced oxidative stress.

Our study showed that concomitant administration of vitamins C and E increased apoptotic changes in villous explants and cytotrophoblastic cells under HR. These results are contradictory to previous reports [28], [29] in which vitamins C (2 mM) and E (1 mM) reduced apoptosis and the secretion of proinflammatory cytokines in villous explants under HR (1 hour at 0.5% O_2_ with subsequent reoxygenation at 10% O_2_ for 16 to 19 hours). The discrepancy may be caused by the differences in the experimental settings: we preconditioned villous explants and cytotrophoblastic cells to HR for 48 hours before administration of vitamins C and E, incubated the explants and cells at different oxygen concentrations for a longer period and used lower concentrations of vitamins C and E. Actually, the effects of vitamins C and E on apoptosis in the human placenta, including fetal membranes, are confusing. It has been shown that vitamin C at 1 mM fails to inhibit hydrogen peroxide-induced apoptosis but instead exacerbates it in epithelial and mesenchymal cells and amnion explants [30]. Using term villous cytotrophoblast preparations, Aris and colleagues found that high levels of vitamins C (50 and 100 µM) and E (20 and 50 µM) affect placental function and immunity in standard culture conditions, as reflected by a decreased secretion of hCG and an increased production of TNF-alpha in a dose-dependent manner [31]. Nevertheless, Tannetta and co-workers showed that administration of 50 µM of vitamins C and E blocks mitochondria-induced apoptosis and syncytiotrophoblast particle shedding and significantly increases hCG secretion into the medium in cytotrophoblastic cells kept at a constant oxygen concentration (10% O_2_) [32].

Although we found there are differential effects of combined administration of vitamins C and E on trophoblast apoptosis and autophagy between normoxia and HR, two limitations of this study merit attention. First, the HR condition in this work is not totally physiological, as the human placenta appears unlikely to be subjected to extreme oxygen tensions for such a long period. Therefore, the results generated by this study should be interpreted with caution. Second, the extrapolation from apoptosis to preterm PROM and fetal demise is still far from proven in this setting. Further studies are required to clarify the effects of high concentrations of vitamins C and E on trophoblast proliferation, death and function.

## References

[1] Hubel CA (1999). Oxidative stress in the pathogenesis of preeclampsia.. Proc Soc Exp Biol Med.

[2] Rumbold AR, Crowther CA, Haslam RR, Dekker GA, Robinson JS (2006). Vitamins C and E and the risks of preeclampsia and perinatal complications.. N Engl J Med.

[3] Poston L, Briley AL, Seed PT, Kelly FJ, Shennan AH (2006). Vitamin C and vitamin E in pregnant women at risk for pre-eclampsia (VIP trial): randomised placebo-controlled trial.. Lancet.

[4] Spinnato JA, Freire S, Pinto ESJL, Cunha Rudge MV, Martins-Costa S (2007). Antioxidant therapy to prevent preeclampsia: a randomized controlled trial.. Obstet Gynecol.

[5] Spinnato JA, Freire S, Pinto ESJL, Rudge MV, Martins-Costa S (2008). Antioxidant supplementation and premature rupture of the membranes: a planned secondary analysis.. Am J Obstet Gynecol.

[6] Villar J, Purwar M, Merialdi M, Zavaleta N, Thi Nhu NN (2009). World Health Organisation multicentre randomised trial of supplementation with vitamins C and E among pregnant women at high risk for pre-eclampsia in populations of low nutritional status from developing countries.. BJOG.

[7] Xu H, Perez-Cuevas R, Xiong X, Reyes H, Roy C (2010). An international trial of antioxidants in the prevention of preeclampsia (INTAPP).. Am J Obstet Gynecol.

[8] Khong TY, De Wolf F, Robertson WB, Brosens I (1986). Inadequate maternal vascular response to placentation in pregnancies complicated by pre-eclampsia and by small-for-gestational age infants.. Br J Obstet Gynaecol.

[9] Hung TH, Burton GJ (2006). Hypoxia and reoxygenation: a possible mechanism for placental oxidative stress in preeclampsia.. Taiwan J Obstet Gynecol.

[10] Crocker IP, Cooper S, Ong SC, Baker PN (2003). Differences in apoptotic susceptibility of cytotrophoblasts and syncytiotrophoblasts in normal pregnancy to those complicated with preeclampsia and intrauterine growth restriction.. Am J Pathol.

[11] Smith SC, Baker PN, Symonds EM (1997). Increased placental apoptosis in intrauterine growth restriction.. Am J Obstet Gynecol.

[12] Tanir HM, Sener T, Artan S, Kaytaz B, Sahin-Mutlu F (2005). Programmed cell death (apoptosis) in placentas from normal pregnancy and pregnancy complicated by term (t) and preterm (p) premature rupture of membranes (PROM).. Arch Gynecol Obstet.

[13] Almog B, Fainaru O, Gamzu R, Kupferminc MJ, Sasson R (2002). Placental apoptosis in discordant twins.. Placenta.

[14] Oh SY, Choi SJ, Kim KH, Cho EY, Kim JH (2008). Autophagy-related proteins, LC3 and Beclin-1, in placentas from pregnancies complicated by preeclampsia.. Reprod Sci.

[15] Shen ZY, Li EM, Lu SQ, Shen J, Cai YM (2008). Autophagic and apoptotic cell death in amniotic epithelial cells.. Placenta.

[16] Levine B, Yuan J (2005). Autophagy in cell death: an innocent convict?. J Clin Invest.

[17] Hung TH, Skepper JN, Burton GJ (2001). In vitro ischemia-reperfusion injury in term human placenta as a model for oxidative stress in pathological pregnancies.. Am J Pathol.

[18] Hung TH, Skepper JN, Charnock-Jones DS, Burton GJ (2002). Hypoxia-reoxygenation: a potent inducer of apoptotic changes in the human placenta and possible etiological factor in preeclampsia.. Circ Res.

[19] Hung TH, Charnock-Jones DS, Skepper JN, Burton GJ (2004). Secretion of tumor necrosis factor-{alpha} from human placental tissues induced by hypoxia-reoxygenation causes endothelial cell activation in vitro: A potential mediator of the inflammatory response in preeclampsia.. Am J Pathol.

[20] Hung TH, Chen SF, Liou JD, Hsu JJ, Li MJ (2008). Bax, Bak and mitochondrial oxidants are involved in hypoxia-reoxygenation-induced apoptosis in human placenta.. Placenta.

[21] Levine B, Sinha S, Kroemer G (2008). Bcl-2 family members: dual regulators of apoptosis and autophagy.. Autophagy.

[22] Chan AC (1993). Partners in defense, vitamin E and vitamin C.. Can J Physiol Pharmacol.

[23] Chappell LC, Seed PT, Briley AL, Kelly FJ, Lee R (1999). Effect of antioxidants on the occurrence of pre-eclampsia in women at increased risk: a randomised trial.. Lancet.

[24] Hung TH, Chen SF, Hsieh CC, Hsu JJ, Li MJ (2008). Tumor necrosis factor-alpha converting enzyme in the human placenta throughout gestation.. Reprod Sci.

[25] Humphrey RG, Sonnenberg-Hirche C, Smith SD, Hu C, Barton A (2008). Epidermal growth factor abrogates hypoxia-induced apoptosis in cultured human trophoblasts through phosphorylation of BAD Serine 112.. Endocrinology.

[26] Siman CM, Sibley CP, Jones CJ, Turner MA, Greenwood SL (2001). The functional regeneration of syncytiotrophoblast in cultured explants of term placenta.. Am J Physiol Regul Integr Comp Physiol.

[27] Crocker IP, Tansinda DM, Baker PN (2004). Altered cell kinetics in cultured placental villous explants in pregnancies complicated by pre-eclampsia and intrauterine growth restriction.. J Pathol.

[28] Tjoa ML, Cindrova-Davies T, Spasic-Boskovic O, Bianchi DW, Burton GJ (2006). Trophoblastic oxidative stress and the release of cell-free feto-placental DNA.. Am J Pathol.

[29] Cindrova-Davies T, Spasic-Boskovic O, Jauniaux E, Charnock-Jones DS, Burton GJ (2007). Nuclear factor-kappa B, p38, and stress-activated protein kinase mitogen-activated protein kinase signaling pathways regulate proinflammatory cytokines and apoptosis in human placental explants in response to oxidative stress: effects of antioxidant vitamins.. Am J Pathol.

[30] Kumar D, Moore RM, Elkhwad M, Silver RJ, Moore JJ (2004). Vitamin C exacerbates hydrogen peroxide induced apoptosis and concomitant PGE2 release in amnion epithelial and mesenchymal cells, and in intact amnion.. Placenta.

[31] Aris A, Leblanc S, Ouellet A, Moutquin JM (2008). Detrimental effects of high levels of antioxidant vitamins C and E on placental function: considerations for the vitamins in preeclampsia (VIP) trial.. J Obstet Gynaecol Res.

[32] Tannetta DS, Sargent IL, Linton EA, Redman CW (2008). Vitamins C and E inhibit apoptosis of cultured human term placenta trophoblast.. Placenta.

